# Développement d’un programme multidisciplinaire de diagnostic de l’adrénoleucodystrophie liée à l’X au Maroc: résultats de la mise en œuvre du programme de diagnostic clinique et biologique

**DOI:** 10.11604/pamj.2017.28.185.11086

**Published:** 2017-10-30

**Authors:** Fatima-Zohra Madani Benjelloun, Layachi Chabraoui, Yamna Kriouile

**Affiliations:** 1 Unité de Neuropédiatrie, Service de Pédiatrie II, Hôpital d’Enfants de Rabat, Maroc; 2 Laboratoire de Biochimie, Centre d’Etude des Maladies Héréditaires du Métabolisme, CHU Ibn Sina Rabat, Maroc; 3 Faculté de Médecine et de Pharmacie de Rabat, Maroc

**Keywords:** Adrénoleucodystrophie liée à l´X, diagnostic, acides gras à très longue chaine, X-linked adrenoleukodystrophy, diagnosis, long-chain saturated fatty acids

## Abstract

**Introduction:**

L'adrénoleucodystrophie liée à l'X (X-ALD) est une maladie neurodégénérative sévère, due à des mutations du gène ABCD1. Elle se manifeste par une atteinte du système nerveux central et périphérique, une insuffisance surrénalienne et une atteinte des testicules chez le garçon. Son diagnostic repose sur le dosage des Acides Gras à Très Longue Chaine. Le diagnostic précoce est d'une grande importance puisque il définit l'accessibilité aux traitements selon le stage de la maladie.

**Méthodes:**

Nous avons mis en place un programme de diagnostic de l'X-ALD au Maroc au niveau de l'Hôpital d'enfants et du Laboratoire centrale des maladies héréditaires et du métabolisme de Rabat. Le programme s'articule sur trois axes à savoir : le recrutement des patients, le diagnostic et la sensibilisation. Le diagnostic s'effectue selon trois protocoles : un protocole pour les cas symptomatiques, un deuxième pour les cas asymptomatiques et un troisième pour les femmes hétérozygotes.

**Résultats:**

Durant trois ans après la mise en place de notre programme de diagnostic de l'Adrénoleucodystrophie liée à l'X, nous avons diagnostiqué la maladie chez sept familles, avec neuf garçons et trois femmes hétérozygotes. Tous les enfants diagnostiqués présentaient la forme cérébrale démyélinisante. Toutes les femmes hétérozygotes étaient asymptomatiques. Une prise en charge thérapeutique a été mise place selon la symptomatologie de chaque cas.

**Conclusion:**

l'X-ALD est une maladie rare. Notre programme de diagnostique a permis de diagnostiquer un nombre important de cas, ce qui montre son importance. Les compagnes de sensibilisation auprès des professionnels permettront de mieux comprendre la maladie et mieux la diagnostiquer et ainsi donner accès à un nombre plus élevé de patients.

## Abstract (en)

**Introduction:**

X-linked adrenoleukodystrophy (X-ALD) is a severe neurodegenerative disease, due to mutations in the ABCD1 gene. It manifests as a damage to the central and peripheral nervous system, adrenal insufficiency and testicular damage in children. Diagnosis is based on the determination of long-chain saturated fatty acids. Early diagnosis is essential because it defines treatment accessibility according to disease stage.

**Methods:**

We implemented a X-ALD diagnostic test program in Morocco at the Children’s Hospital and at the Central Laboratory for inherited and metabolic diseases in Rabat. The program was based around three priorities, namely: the recruitment of patients, diagnosis and awareness. Diagnosis is based on three protocols: a protocol for symptomatic cases, a protocol for asymptomatic cases and a protocol for heterozygous women.

**Results:**

During the first three years after implementation of our X-ALD diagnostic test program, we diagnosed the disease in seven families, with nine boys and three heterozygous women. All children were diagnosed with demyelinating brain. All heterozygous women were asymptomatic. Different symptom-based therapies were established.

**Conclusion:**

X-ALD is a rare disease. Our diagnostic program has helped to diagnose a significant number of cases, hence its importance. Campaigns focused on raising awareness among health care professionals will enable a better understanding of the disease and a more accurate diagnosis as well as to improve access to health care for a higher number of patients.

## Introduction

L'adrénoleucodystrophie liée à l'X (X-ALD) est une maladie neurodégénérative sévère [1] qui associe une démyélinisation progressive du système nerveux central et périphérique, une insuffisance surrénalienne (maladie d'Addison) et une insuffisance gonadique [2]. Avec une incidence de 1:17 000, elle constitue la maladie la plus commune des leucodystrophies et des maladies peroxysomales [3]. L'X-ALD est due à des mutations du gène ABCD1 (ATP-Binding Cassette-membre1) [4], qui code pour la protéine ALDP, un hémi-transporteur transmembranaire au niveau du peroxysome, ou s'opère la beta oxydation des acides gras à très longue chaine (AGTLC) [5]. On distingue trois principales formes d'expression clinique de la maladie: les formes cérébrales démyélinisantes de l'enfant, la forme médullaire ou l'adrénomyéloneuropathie (AMN) qui touche l'homme adulte et 35% des femmes hétérozygotes et la maladie d'Addison seule sans signes neurologiques [1,2,6,7]. Cette variabilité d'expression clinique ainsi que la non corrélation phénotype-génotype rendent le diagnostic de la maladie très difficile dans la plupart des cas [8,9]. Le traitement de la maladie à l'état actuel se base essentiellement sur la greffe allogénique de moelle osseuse, à condition qu'elle soit opérée à un stade très précoce de la maladie, au stade asymptomatique [10]. Tout ceci, fait que le maillon « Diagnostic », constitue un maillon très critique pour cette maladie [11]; aussi, le conseil génétique est d'une grande importance, et permet de détecter les cas asymptomatiques et les femmes hétérozygotes qui risquent de transmettre la maladie à leur progéniture [12]. Nous avons mis en place un programme multidisciplinaire de diagnostic de X-ALD, incluant l'unité de neuro-pédiatrie à l'hôpital d'enfants de Rabat et le laboratoire central des maladies héréditaires du métabolisme, en collaboration avec différentes structures médicales et paramédicales. L'objectif de cette étude est de donner un état des lieux de la maladie de l'adrénoleucodystrophie au Maroc, en présentant les résultats retrouvés suite à l'installation du programme de diagnostic de l'X-ALD au Maroc.

## Méthodes

Il s'agit d'une étude descriptive transversale, avec recrutement des patients sur une période de 3 ans. Cette étude a été réalisée au niveau des services du centre hospitalier Ibn Sina.

**Création du programme:** Le programme multidisciplinaire de diagnostic de l'X-ALD, est un programme qui est né suite au besoin d'effectuer le diagnostic précoce de la maladie chez la population marocaine. Il a été créé en collaboration entre deux principales structures médicales du Centre Hospitalier Universitaire (CHU) Ibn Sina de Rabat, qui sont l'unité de neuro-pédiatrie de l'hôpital d'enfants et le Laboratoire de Biochimie du Centre d'étude des maladies héréditaires du métabolisme à Rabat. Le programme comporte trois principales composantes: 1) le recrutement des patients; 2) Le diagnostic clinique et biologique; 3) La sensibilisation du personnel médical par rapport à la maladie. Ainsi, les patients sont recrutés à partir des consultations de l'unité de neuro-pédiatrie, suite à un tableau clinique évocateur que nous avons élaboré dans une fiche décrivant les signes cliniques évocateurs (Tableau 1). Avant de rejoindre le protocole de diagnostic, les patients affirment par écrit leur accord à y adhérer en signant une fiche de consentement. La fiche est rédigée en langue arabe avec une traduction en langue française, pour qu'elle soit explicite et compréhensible par le lecteur et le signataire. Vu que la maladie prend plusieurs formes cliniques, nous avons conçu trois protocoles de diagnostic. La sensibilisation cible le personnel du service de neuro-pédiatrie, en expliquant la maladie, les symptômes évocateurs dressés sur le Tableau 1, le diagnostic et le suivi de la maladie, ainsi que les traitements disponibles à l'état actuel. Une sensibilisation plus large au profit des acteurs dans le domaine de la santé a été effectuée à travers des conférences, des communications orales lors des journées scientifiques et des congrès médicaux, et aussi à travers des brochures de sensibilisation que nous avons mise en œuvre. Il est à signaler que le comité d'éthique pour la Recherche Biomédicale (CERB) de l'université Mohammed V a donné son accord pour la réalisation de ce projet.

**Tableau 1:** Signes cliniques évocateurs de l’X-ALD

**Symptômes neurologiques**	
	Neuro-régression
	Spasticité
	Ataxie
	Paraparésie spastique
	Atteinte de la vision et l’audition
**Mélanodermie**	Coloration des conjonctives, des lèvres et des extrémités digitées
**Symptômes digestifs**	
	Vomissements
	Diarrhées
**Troubles de comportement**	
	Hyperactivité
	Nervosité
**Troubles cognitifs**	
**Diminution des performances scolaires**	

**Figure 1:**
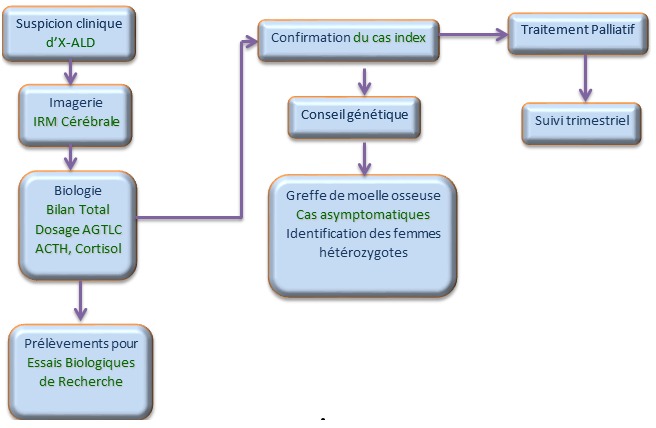
Schéma de diagnostic et de suivi de l'X-ALD

**Protocole général de diagnostic :** Lors d'une suspicion de la maladie nous suivons un schéma de confirmation selon un protocole bien défini, qui repose sur l'imagerie par résonnance magnétique (IRM), des examens biochimiques pour l'exploration de la fonction surrénale, à savoir le dosage du cortisol et de l'ACTH et un dosage des AGTLC qui constituent le biomarqueur de la maladie (Figure 1). Après la confirmation de la maladie chez le cas index, une réunion est organisée avec les parents ou les responsables de l'enfant malade afin de leur expliquer la maladie, dresser l'arbre généalogique, et signer les consentements pour le conseil génétique, ce qui permet de repérer les patients asymptomatiques et les femmes hétérozygotes de la famille.

**Figure 2:**
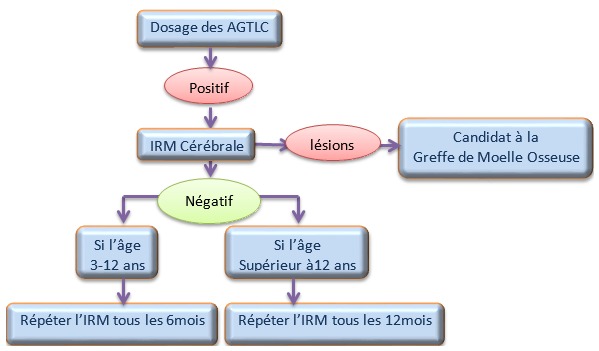
Schéma du protocole de suivi des cas asymptomatiques

**Protocole des cas asymptomatiques:** Les enfants asymptomatiques, après confirmation par dosage des AGTLC, sont suivis périodiquement tous les six mois par IRM à partir de l'âge de quatre ans, afin de suivre l'évolution des lésions de démyélinisation. Dès l'apparition des premières lésions qui évoluent, le patient est transféré aux services spécialisés dans la greffe allogénique de moelle osseuse afin d'étudier la possibilité et la faisabilité de bénéficier de ce traitement (Figure 2).

**Protocole des femmes hétérozygotes:** Les femmes hétérozygotes, une fois confirmées atteintes d'X-ALD, une fiche signalétique leur est attribuée contenant toutes leurs informations. Tous les risques relatifs à la transmission de la maladie à leur progéniture leur sont expliqués, ainsi que les moyens de prévention possibles, en particulier le diagnostic préimplantatoire. Les femmes hétérozygotes dépassant quarante ans, sont suivies annuellement pour détecter les premiers symptômes d'une éventuelle adrénomyéloneuropathie, et délivrer les traitements adaptés le cas échéant.

## Résultats

**Figure 3:**
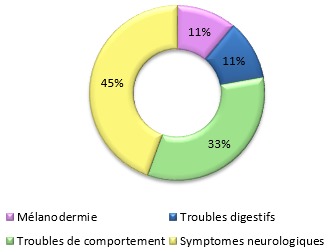
Motifs de consultations lors d'une atteinte d'adrénoleucodystrophie liée à l'X

Après trois ans de mise en marche du programme, nous avons diagnostiqué l'adrénoleucodystrophie chez sept familles, avec neuf garçons et trois femmes hétérozygotes. Le Tableau 2 donne un résumé des cas diagnostiqués durant ces trois ans. Chez les enfants recrutés, le motif de consultation est principalement l'apparition de symptômes neurologiques, les troubles de comportement viennent en deuxième position, et en troisième position les symptômes de l'insuffisance surrénalienne (Figure 3). Tous les enfants diagnostiqués avaient la forme cérébrale démyélinisante. Le Tableau 3, le Tableau 4 et le Tableau 5 montrent les principaux symptômes retrouvés chez les patients su le plan neurologique, endocrinologique et comportementale. Au cours de cette étude nous avons perdu 4 enfants qui sont morts dans un état grabataire, le Tableau 6 décrit les principaux symptômes retrouvés lors de la phase finale de la maladie. Sur le plan paraclinique, l'IRM cérébrale montre une anomalie de signal de la substance blanche pariéto-occipitale bilatérale chez 7 garçons, une atrophie cérébelleuse chez un garçon, et une Atrophie vermineuse des deux hémisphères cérébraux chez un seul garçon. L'atteinte par l'X-ALD est confirmée par le dosage des AGTLC, les rapports des acides gras C24/C22 et C26/C22 des cas étudiés sont représentés dans le Tableau 7.

**Tableau 2:** Résumé des cas diagnostiqués au cours du projet

Famille	Nombre cas	Sexe	Age	Etat
**1**	5	1 garçon	10	Mort
		1 garçon	15	Malade
		1 garçon	10	Mort
		1 F. hétérozygote	29	Asymptomatique
		1 F. hétérozygote	27	Asymptomatique
**2**	1	1 garçon	9	Mort
**3**	1	1 garçon	5	Mort
**4**	1	1 garçon	8	Malade
**5**	1	1 garçon	12	Malade
**6**	1	1 garçon	12	Malade
**7**	1	1 garçon	9	Malade
**Total**	11	-	-	-

**Tableau 3:** Principaux symptômes neurologiques retrouvés chez les enfants

Symptômes neurologiques	Nombre de cas	Fréquence (%)
**Ataxie cérébelleuse**	1	11
**Paraparésie**	4	44
**Syndrome pyramidal**	5	55
**Hémiplégie**	4	44
**Céphalées**	4	44
**Démence**	1	11

**Tableau 4:** Les symptômes de l’insuffisance surrénalienne retrouvés chez les garçons

Symptômes de l’insuffisance surrénalienne	Nombre de cas	Fréquence %
**Mélanodermie**	9	100
**Troubles digestifs**	7	77
**Douleurs abdominales**	7	77

**Tableau 5:** Principaux symptômes comportementaux chez les enfants

Troubles de comportement	Nombre de cas	Fréquence %
**Déficit de la mémoire immédiate**	2	22
**Repliement sur soi**	4	44
**nervosité**	5	55
**excitabilité**	2	22
**Diminution des performances scolaires**	9	100
**Troubles de l’attention**	8	88
**Troubles émotionnels**	1	11

**Tableau 6:** Principaux symptômes lors de la phase finale de la maladie

Symptôme	Nombre cas	Age moyen d’apparition
**Cécité**	3	7.5
**surdité**	3	5
**Tétraplégie**	4	7.5
**Démence**	1	7
**Mort**	4	7.5

**Tableau 7:** Résultats du dosage des acides gras à très longue chaine chez les garçons

Cas	C24/C22 N : 0.665-1.008	C26/C22 N : 0.005-0.025
**1**	1,35	0,061
**2**	2.05	0.130
**3**	0,97	0,051
**4**	1,87	0,081
**5**	2	0,102
**6**	1,33	0,09
**7**	1.01	0.054
**8**	1.230	0.073
**9**	1.81	0.068

Le dosage du cortisol et de l'ACTH sont effectués dans un objectif de diagnostic et de traitement, afin de confirmer l'atteinte de la fonction surrénalienne et instaurer un traitement substitutif par hydrocortisone. La cortisolémie était inférieurs aux normes chez 67% des cas et dans les normes dans 33% des cas. Chez les enfants ayant une cortisolémie inférieures aux normes, on retrouve des valeurs très élevés de l'ACTH ce qui confirme l'atteinte périphérique. Lors des conseils génétiques qui succèdent le diagnostic du cas index, nous avons diagnostiqué trois femmes hétérozygotes. Toutes ces femmes étaient asymptomatiques, leurs âge est respectivement 25, 27 et 45 ans. Nous avons constaté que l'âge moyen d'apparition du premier symptôme est de 6 ans, alors que la première consultation au niveau de nos services se fait vers un âge moyen de 8 ans, ce qui représente un intervalle de temps de deux ans entre l'apparition du premier symptôme et la consultation. Ce qui nous a menés à effectuer des actions de sensibilisation. Ainsi, nous avons participé à 9 manifestations scientifiques en présentant la maladie, son statut actuel au Maroc, ses répercussions sur le malade et sa famille et les perspectives de nos recherches sur ladite pathologie. Nous utilisons aussi des supports tels que les posters et des brochures qui sont distribués lors de ces évènements. Ces présentations sont destinées aux professionnels de la santé, à savoir les médecins, les pharmaciens, les biologistes et les chercheurs de différents aspects médicaux. Nous avons reçu plusieurs demandes d'équipes médicales souhaitant avoir plus d'informations sur la maladie et sur notre projet de recherche.

## Discussion

L'étude que nous avons menée, est une étude descriptive transversale sur trois ans, ayant inclus sept familles, avec neuf garçons et trois femmes hétérozygotes. En consultant le registre de l'hôpital d'enfants de Rabat, on s'est aperçu que durant 5 ans avant l'installation de notre programme, il n'y avait que 5 cas étiquetés suspects d'Adrénoleucodystrophie. Deux enfants seulement ont été confirmés est diagnostiqués par dosage des AGTLC. Après la mise en place de notre programme, nous avons diagnostiqué 11 cas confirmés au bout de 3 ans. Ce qui montre l'importance et l'apport significatif de notre projet. Le diagnostic de ces cas confirmés atteints d'X-ALD, a permis d'offrir un traitement symptomatique palliatif des atteintes neurologiques qui sont présents chez tous les enfants atteints, et a permis d'offrir un traitement de substitution pour l'insuffisance surrénalienne, qui est présente chez 88% des garçons malades. Toutes ces actions instaurées avaient comme conséquences le soulagement des douleurs des patients et l'amélioration de leur état de santé et leur bien-être. Aussi notre programme a permis d'assurer un suivi et un accompagnement au cours de la phase finale pour les enfants qui sont morts au cours de cette étude. Le suivi trimestriel, permet de suivre l'évolution de la maladie [13], de détecter à temps les nouveaux symptômes sur le plan neurologique et d'offrir le traitement palliatif et l'ajuster à chaque étape de l'évolution [14]. L'identification de femmes hétérozygotes offre une opportunité de prévention de la maladie via le conseil génétique [15]. Les résultats négatifs de dosage des AGTLC sont combinés à la recherche de mutation pour avoir une confirmation et une assurance du statut sain de la femme à risque. Une telle information apporte un grand soulagement pour les femmes recensées à risque suite à l'étude de l'arbre généalogique. Cependant, dans le cas de l'annonce d'une femme hétérozygotes, plusieurs questions se posent par la femme et son mari, et qui sont toujours en relation avec la transmission de la maladie à la progéniture et les moyens de prévention.

le statut asymptomatique s'explique par leur âge, mais il est à signaler qu'elles sont candidates à à développer la forme médullaire, l'adrénomyéloneuropathie, dans le futur, vers l'âge de 50ans [1,16]. Le conseil génétique est très productif et la communication est toujours fructueuse. En effet, les membres des familles adhèrent pleinement au programme du fait qu'il s'agit d'une maladie rare et difficile à vivre tout seul. L'adrénoleucodystrophie, en plus de son impact sanitaire, a d'autres impacts psychologiques et sociaux [9]. Nous avons constaté suite à ce programme que l'X-ALD à un impact dévastateur sur la famille dans laquelle on diagnostique un ou plusieurs garçons atteints. En effet, dès la déclaration d'un cas index dans la famille, toute l'attention, le temps et les ressources financiers, sont dirigés vers le management du nouveau problème, qui nécessite du temps et de la patience pour suivre les différentes étapes du programme allant de la confirmation de la maladie chez le cas index, le conseil génétique, le suivi des nouveaux cas diagnostiqués, jusqu'à l'installation des traitement adaptés. L'expérience a montré que c'est pendant ce moment il faut bien maitriser la communication avec les membres de la famille et les parents en premier et particulièrement la maman de l'enfant atteint. L'intervalle de temps entre la première consultation dans nos services et l'apparition du premier symptôme qui est de deux ans, est un intervalle important pendant lequel les parents des enfants malades affirment qu'ils ont consulté chez plusieurs médecins et équipes médicales sans aboutir au bon diagnostic, ce qui renforce la nécessité d'effectuer la sensibilisation vis-à-vis de la maladie à plus grande échelle chez les professionnels de la santé et les structures médicales. Ceci s'intègre dans la nécessité d'effectuer un diagnostic précoce afin d'augmenter l'accessibilité aux thérapies adaptées [17].

## Conclusion

Le programme de diagnostic et de suivi de l'adrénoleucodystrophie mis en place par l'équipe multidisciplinaire de l'hôpital d'enfant de Rabat et le centre de diagnostic des maladies héréditaire et du métabolisme est le premier programme qui s'intéresse à cette maladie au Maroc. Sa valeur ajoutée est démontrée par le nombre de cas diagnostiqués et par le soulagement des souffrances des familles atteintes en offrant un suivi régulier et des traitements adaptés, et aussi par l'accompagnement et la communication continue en répondant à toutes les questions et les besoins spécifiques à tout moment. Ce programme a donné naissance à une équipe de recherche sur l'adrénoleucodystrophie qui travaille sur les aspects biochimiques et moléculaires de la maladie, et qui a commencé à faire sortir les premiers résultats de mise au point de techniques biochimiques et moléculaire de diagnostic de la maladie qui seront publiés dans une prochaine étude.

### Etat des connaissances actuelle sur le sujet

- L'adrénoleucodystrophie liée à l'X est une maladie neurodégénérative rare;
- Le diagnostic repose sur le dosage des acides gras à très longue chaine;
- Le diagnostic précoce est d'une grande importance puisqu'il permet l'accès au traitement.

### Contribution de notre étude à la connaissance

- L'installation d'un premier programme multidisciplinaire de diagnostic de l'adrénoleucodystrophie liée à l'X au Maroc;
- Description des profils cliniques et biologiques des patients atteints de l'adrénoleucodystrophie au Maroc;
- Sensibilisation des professionnels de santé par rapport à la maladie pour éviter les erreurs de diagnostic et permettre un diagnostic précoce aux patients.

## Conflits d’intérêts

Les auteurs ne déclarent aucun conflit d'intérêts.
